# Multi-level repression ensures tight translation control of C9ORF72

**DOI:** 10.1101/2025.08.09.669471

**Published:** 2026-10-22

**Authors:** Solomon A. Haizel, Alfredo Vidal Ceballos, Alice Nayoung Ko, Lucas Lyu, Lauren Manning, Xinyi Ge, Violet Park, Huijing Zou, Churong Cheng, Richmond Afful, Serena Kher, Anthony Widjaja, Joanetta Jean-Baptiste, Sasha Khazhinsky, Pieter Spealman, Alisha N. Jones, Leos Shivaya Valasek, Christine Vogel

## Abstract

C9ORF72 is involved in multiple neuronal functions and a major factor in Amyotrophic Lateral Sclerosis, a fatal neurodegenerative disease. Pathogenicity is implemented through an intronic repeat expansion in the gene’s mRNA leader that produces long, repetitive RNA and dipeptides when translated through repeat associated non-canonical translation. The expanded repeat also suppresses transcription of the major transcript variant (Variant 2) which comprises 90% of the C9ORF72 levels in healthy cells, leading to an additional loss-of-function effect. Examining the sequence, structure, and ribosome occupancy of Variant 2’s mRNA leader in detail, we found that translation of C9ORF72 is tightly regulated through a multi-layered system. To deconvolute this system, we conducted a mutant screen and developed a method to evaluate the results quantitatively. We found that near-cognate start codons in all three frames, a start-stop element in frame 2, a uTranslon in frame 1, and secondary structures cooperate to keep baseline C9ORF72 translation low. Further, initiation at upstream near-cognate start codons reduces the impact of both the start-stop and the uTranslon, effectively buffering their repressive function and creating a feedforward loop. A strong secondary structure in the Variant 2-specific sequence further modulates initiation at all the elements. We hypothesize that this buffered repressor system has evolved to ensure reliable, noise-insensitive expression of this critical regulator of neuronal function. Finally, we used ADAR-based RNA editing of specific sites in Variant 2’s mRNA leader, increasing translation of the gene and providing a novel avenue to rescue the loss-of-function phenotype in disease.

